# Prediction of Antidepressant Efficacy by Cognitive Function in First-Episode Late-Life Depression: A Pilot Study

**DOI:** 10.3389/fpsyt.2022.916041

**Published:** 2022-05-20

**Authors:** Weigang Pan, Chaomeng Liu, Dandi Zhu, Yi Liu, Peixian Mao, Yanping Ren, Xin Ma

**Affiliations:** ^1^The National Clinical Research Center for Mental Disorders and Beijing Key Laboratory of Mental Disorders, Beijing Anding Hospital, Capital Medical University, Beijing, China; ^2^Advanced Innovation Center for Human Brain Protection, Capital Medical University, Beijing, China

**Keywords:** treatment response, remission, aging population, major depression (MDD), cognitive predictors, cognitive function

## Abstract

The response rate of treatment for late-life depression (LLD) is only 25–60%. The cognitive impairment associated with LLD often affects the effectiveness of antidepressants and may has the potential ability to predict response. This study seeks a biomarker for baseline cognitive function to predict efficacy of antidepressants. Sixty patients diagnosed with LLD received escitalopram or sertraline treatment for 8 weeks. Clinical symptom was measured using Hamilton Depression Rating Scale-17 (HAMD-17) and cognitive function was measured using the Repeatable Battery for the Assessment of Neuropsychological Status (RBANS), Trail Making Test (TMT) before and after 8-week treatment. Patients were divided into treatment effective group (TE) and treatment ineffective group (TI) according to reduction rate in scores of HAMD-17 after treatment. Thirty-eight matched healthy controls (HC) were assessed using RBANS and TMT. There was significant decrease of score of RBANS and increase of score of TMT in patients with LLD compared with HC. Regression analysis revealed that change in HAMD-17 score was significantly positively associated with baseline score of picture naming, figure copy, digit span, and delayed memory. The preliminary findings suggested that working memory, attention, visuospatial, language function, and delayed memory should be examined further as a means of providing the useful objective biomarkers of treatment response.

**Clinical Trials Registration:**

[www.ClinicalTrials.gov], identifier [ChiCTR2100042370].

## Introduction

Late-life depression (LLD) can be defined as major depressive disorder that occurs for 60 years of age or older (1, 2). The global prevalence of LLD is 13.3%, which is significantly higher than that of depression in younger age (3). The response rate of treatment for late-life depression (LLD) is only 25–60% (4). Because treatment effects may be delayed during antidepressant treatment, the guideline continues to recommend 4–6 weeks of treatment until treatment failure. This approach to medication selection contributes to treatment failure and unnecessarily exposes patients to lengthy and inadequate treatment trials, prolonging patient morbidity (5). Identifying predictors of early efficacy for antidepressants is an important issue to be solved and have great clinical significance because it will enable clinicians to determine as early as possible whether patients will benefit from specific types of treatment (4, 5). In addition, the prediction of antidepressant efficacy could improve treatment sensitivity, which would help reduce unnecessary drug exposure (6). Treatment-resistant depression is detected promptly and antidepressant therapy can be optimized as early as possible (7) which could improve the patient’s quality of life, reduce the medical burden, and even reduces the risk of suicide of patients (8).

Cognitive dysfunction, such as decreased ability to think and concentrate, and difficulty in making decisions, is one of the main clinical manifestations of depression and a diagnostic item in the Diagnostic and Statistical Manual of Mental Disorders, fifth edition (DSM-5) (9). LLD has a particularly prominent cognitive impairment compared with youth depression (10). Cognitive symptoms in LLD are mainly manifested as functional impairments in executive function, memory, and information processing speed, which are risk factors affecting social functional outcomes (5, 10, 11). There was discrepancy of the relationship for cognitive function and response to antidepressant therapy in LLD. Several studies suggested that cognitive symptoms appeared in the acute periods of depression, which would affect the effective rate of antidepressant treatment (12, 13). A recent meta-analysis investigated the relationship between antidepressant efficacy in LLD and attention, suggesting that executive function deficits in LLD are associated with poor prognosis (5). It is generally believed that the impairment of executive function in LLD affects the efficacy of antidepressants (10). Morimoto et al. (14) found that baseline TMT scores (indicative of greater executive dysfunction) predicted percent change of Montgomery Asberg Depression Rating Scale (MADRS) over the 4 weeks in LLD. The result appeared to distinguish verbal perseveration from verbal initiation as the cognitive process that was most associated with poor treatment response. Similarly, in the largest positive study, Potter et al. (15) found that perseverative responses during verbal initiation tasks that fewer perseverative errors on the Controlled Oral Word Association Task and better performance on Digit Span significantly predicted better remission status in LLD (*n* = 110). Another meta-analysis (17 studies) analyzed the prediction of antidepressant efficacy by different cognitive impairments (90 cognitive assessment tools) found that impairment of working memory and delayed recall were associated with poor antidepressant efficacy (16). This finding was also reported by Sheline et al. (17), who found that baseline episodic memory, language, working memory predicted percent change of MADRS in LLD. In accordance with the present results, previous study had demonstrated that best prose recall at baseline exhibited the greatest treatment response at follow-up (18). It was inconsistent about the relationship of cognitive function impairment and antidepressant efficacy in LLD. Pimontel et al. (4) concluded from a meta-analysis of cognitive testing in LLD, that only planning and organization (measured by a subtest of the Dementia Rating Scale) were associated with antidepressant efficacy. Other studies have found that there was no association between executive function and antidepressant efficacy (19–21). Several other studies have suggested no correlation between verbal learning and memory performance in LLD and antidepressant efficacy (22–24).

Although there is emerging evidence indicating that there might be potential cognitive function to predict treatment response in LLD, conclusions from these studies are limited by the using of unequally treatment, inadequate follow-up time and frequency, small sample size subjects, and only assessed one or a small number of cognitive domains, which resulting in inconsistent information to identify the cognitive function on prediction of response of treatment. In addition, some studies included relapsed patients, which is difficult to differentiate the effect on cognition of previous use of antidepressant drugs from the factors of the disease itself (5). Especially, patients with LLD are a heterogeneous group, including individuals with early-onset depression in whom the initial depression manifesting occurs earlier in life, and individuals with late-onset depression who had a first depressive episode after age 60 years (1, 2). Late-onset depression has a particularly prominent cognitive impairment compared with early-onset depression (10). To the best of our knowledge, it is still unclear which cognitive function best predicts antidepressant response of first-episode, drug naive LLD patients.

In this study, we discuss how cognitive dysfunction may contribute to the treatment response in late-onset depression. During this longitudinal study, we measured Trail Making Test A (TMT-A), Trail Making Test B (TMT-B) and Repeatable Battery for the Assessment of Neuropsychological Status (RBANS), to determine whether the baseline measures of these could be used to predict response after 8 weeks of antidepressant treatment. The association between baseline cognitive testing (TMT-A, TMT-B, and RBANS) score and percent change in 17-item Hamilton Depression (HAMD-17) score after antidepressant was assessed.

We hypothesized that LLD was widespread cognitive function impairment affect disease prognosis. The purpose of the current study was to identify cognitive function processes that may be associated with treatment response in LLD. One goal was to further examine the roles of cognitive function as predictor of treatment response. Moreover, we aimed to identify the role of cognitive function, such as immediate memory, delayed memory, visuospatial, language, and executive function, that might influence treatment response in LLD.

## Materials and Methods

### Participants

Eighty LLD patients were recruited from the outpatient clinic of Beijing Anding Hospital, Affiliated Capital Medical University from January 2021 to November 2021. LLD patients met the following inclusion criteria: (1) age ≥ 60 years old, with an education level more than 6 years; (2) first episode of depression occurred after the age of 60; (3) met the criteria for major depression according the Diagnostic and Statistical Manual of Mental Disorders, Fifth Edition (DSM-5); (4) HAMD-17 score ≥ 17; (5) not taking antidepressants when enrollment. Twenty of these patients did not complete the study. Thus, the final analysis consisted of sixty LLD patients. Thirty-eight healthy controls (HC) were recruited from the communities in Beijing, China. The inclusion criteria for HCs as follows: (1) age ≥ 60 years old; (2) no psychiatric disorder, cognitive function is normal; (3) no psychotropic drug treatment. The exclusion criteria of all participants were as follows: (1) participants with previous manic or hypomanic episode; (2) comorbid dementia, psychiatric or medical conditions; (3) serious medical illnesses like cardiovascular, hepatic, renal, etc.; (4) history of brain injury; (5) substance abuse or dependence; (6) score of Minimum Mental State Examination (MMSE) more than 20 for primary education level, or score of MMSE more than 24 for equal to and over middle school education level over middle school (25, 26).

The study was approved by the Ethics Committee of Beijing Anding Hospital, Capital Medical University (2020-Scientific Research-97). All participants or their family members were required to provide written informed consent before entering the study. The trial was registered in the Chinese Clinical Trial Registry (ChiCTR2100042370).

### Antidepressant Treatment and Efficacy Assessment

Patients diagnosed with LLD received 8-week antidepressant treatment with escitalopram or sertraline. The dosage of the drug was adjusted by doctor according the patient’s clinical conditions. Patients with severe sleep disturbance, anxiety or agitation may be treated with short-term benzodiazepines. During the 8-week treatment period, none of the patients received neurostimulation therapy such as electroconvulsive therapy or transcranial magnetic stimulation therapy. The clinical symptom was measured using the HAMD-17 at baseline and 8-week after treatment.

Rates of response was defined as a ≥ 50% reduction in HAM-17 total score from baseline to post 8-week treatment. The percent changes in HAMD-17 were determined using: (baseline HAMD-17– posttreatment HAMD-17)/baseline HAMD-17 ×100%. The patients with LLD were divided into two groups based on change of HAMD-17. Treatment effective group (TE) was defined as the changes in HAMD-17 score 50% or higher and treatment ineffective group (TI) was defined as changes in HAMD-17 score less than 50%.

### Cognitive Function Assessment

TMT and RBANS were used to assess cognitive function. The TMT-A test connects the circles with numbers (1∼25) written in sequence, and the TMT- B test connects the numbers and Chinese characters in an alternating manner. The operation time and error number of TMT-A test reflects the visuospatial scanning and writing ability of the subjects, and the operation time and error number of TMT-B test reflects the ability of the subjects to transform between different sequences. The longer the time spent and the more errors, the lower the cognitive flexibility of the subjects. The test reflects executive function, attention, and information processing speed. RBANS consists of 12 test tasks (12 items) to assess 5 cognitive function indicators (5 factors): Immediate Memory: assessed by list learning and story memory; Visuospatial: composed of figure copy and line orientation; Language: including picture naming and semantic fluency; Attention: consisted of digital span and coding; Delayed Memory: composed of list recall, list recognition, story recall and figure recall. RBANS is easy to perform and takes nearly 20 min to administer. The mean of the RBANS index score and subscale score is 100 in each instance, with the standard deviation of each instance is 15.

LLD patients were assessed for cognitive function at baseline and after 8-week treatment. HC participants completed the cognition test once when entering the trial.

### Statistical Analysis

The data were analyzed using Statistical Package for Social Sciences version 23.0 (IBM SPSS 23.0, Chicago, IL, United States). Data were tested for normality using Kolmogorov-Smirnov test. The non-parametric data were compared by Wilcoxon-Mann-Whitney test. Between the NC group and LLD group, we examined differences in demographics, cognitive testing using *t*-test for continuous variables and χ^2^-test for categorical variables. The difference of change in cognitive testing (TMT-A, TMT-B and RBANS) score and HAMD-17 score between TE and TI group were examined using repeated-measures ANCOVAs. Multiple linear regression analysis was performed to analyze potential impact on response rate of various risk factors including cognitive function, clinical characteristic, and sociodemographic data. Binary logistic regression analysis was performed to analyze the predictors of efficacy. Receiver operating characteristic (ROC) curve was used to evaluate the predictive ability of the model constructed by logistic regression analysis. *p* < 0.05 was considered statistically significant. The significance level was set to *p* < 0.05, two-tailed.

## Results

### Participants

A total of eighty LLD patients and 38 HC were recruited. Twenty of LLD patients did not complete the study. Thus, the final analysis consisted of sixty LLD patients and 38 HC. There were no significant differences in age, gender, and education level between two groups (see Table 1).

**TABLE 1:** The demographic and clinical characteristics of the LLD and HC group.

Variables	LLD (*n* = 60)	HC (*n* = 38)	*t/*χ ^2^-value	*p*-value
Age (years)	67.75 ± 4.732	65.92 ± 4.122	1.957	0.053
Sex (male/%)	19 (31.7%)	15 (39.5%)	0.626	0.429
Education years	10.32 ± 3.311	10.97 ± 1.881	−1.113	0.268

*LLD, late life depression; HC, healthy control.*

The scores of TMT-A (*z* = 4.259, *p* < 0.001) and TMT-B (*z* = 5.042, *p* < 0.001) in LLD group were significantly higher than those in HC group. There were significant differences in immediate memory (*t* = −38.977, *p* < 0.001), visuospatial (*t* = −40.824, *p* < 0.001), language (*z* = −8.319, *p* < 0.001), attention (*t* = −75.350, *p* < 0.001), delayed memory (*t* = −30.671, *p* < 0.001), and RBANS total score (*z* = −4.871, *p* < 0.001) between LLD and HC groups, with the LLD patient group being worse overall (see Table 2).

**TABLE 2:** Comparison of cognitive function between LLD and HC group.

Cognitive task	LLD (*n* = 60)	HC (*n* = 38)	*t/z*-value	*p*-value
TMT-A	54.27 ± 20.98	36.7 ± 11.36	4.259*	< 0.001
TMT-B	95.24 ± 39.3	58.77 ± 19.08	5.042*	< 0.001
RBANS total score	119.34 ± 14.44	147.05 ± 28.97	−4.871*	< 0.001
Immediate memory	24.27 ± 7.81	112.16 ± 14.48	–38.977	< 0.001
List learning	17.23 ± 4.79	24.32 ± 6.19	−5.123*	< 0.001
Story memory	7.08 ± 3.91	10.71 ± 3.76	–4.542	< 0.001
Visuospatial	31.5 ± 8.56	107.61 ± 9.64	–40.824	< 0.001
Figure copy	15.18 ± 3.79	16.97 ± 2.77	−2.726*	0.006
Line orientation	13.37 ± 3.35	16.05 ± 2.61	−3.940*	< 0.001
Language	28.53 ± 5.5	107.39 ± 6.36	−8.319*	< 0.001
Picture naming	9.15 ± 1.49	9.79 ± 0.41	−2.898*	0.004
Semantic fluency	16.8 ± 4.46	22.47 ± 4.05	−5.445*	< 0.001
Attention	25.9 ± 5.15	123.11 ± 7.63	–75.35	< 0.001
Digit span	13.35 ± 2.83	15 ± 1.43	−2.997*	0.003
Coding	23.98 ± 9.61	38.47 ± 10.01	–7.155	< 0.001
Delayed memory	36.8 ± 11.4	105.74 ± 9.88	–30.671	< 0.001
List recall	2.98 ± 4.01	4.68 ± 2.27	−4.246*	< 0.001
List recognition	17.03 ± 1.96	18.84 ± 1.35	−4.955*	< 0.001
Story recall	3.17 ± 2.25	5.42 ± 2.34	−4.213*	< 0.001
Figure recall	8.63 ± 4.87	11.58 ± 4.45	–3.017	< 0.001

LLD, late life depression; HC, healthy control; TMT, trial making test; RBANS, Repeatable Battery for the Assessment of Neuropsychological Status. *z-test.

### Effects of Clinical Symptoms and Impact on Cognitive Function of Antidepressant Treatment in Late-Life Depression Group

In the LLD group, compared with baseline, there was a significant improvement in the HAMD (*t* = 13.925, *p* < 0.001) after 8-week treatment. The reduction rates (%) of HAMD was 58.091 ± 24.495.

Compared with baseline, there were statistically significant differences in immediate memory (*t* = −3.389, *p* = 0.001), language (*t* = −2.160, *p* = 0.035), delayed memory (*t* = −4.946, *p* < 0.001), and RBANS total score (*t* = −5.238, *p* < 0.001) after treatments in the LLD group.

### Comparison of Cognitive Performance Between Treatment Effective Group and Treatment Ineffective Group in Late-Life Depression Group

A total of 39 cases (65%) were responsive (TE) and 21 LLD patients (35%) were non-responsive (TI) after 8-week treatment. There was no significant difference between the TE and TI group in age, gender, education level, age of onset, duration of disease, antidepressant drugs, and baseline scores of HAMD-17 (see Table 3).

**TABLE 3:** The demographic and clinical characteristics of the TE and TI group.

Variables	TE (*n* = 39)	TI (*n* = 21)	*t/*χ ^2^-value	*p*-value
Age (years)	67.79 ± 4.98	67.67 ± 4.35	–0.099	0.921
Sex (male, *n*, /%)	13 (33.3%)	6 (28.6%)	0.143	0.705
Education years	10.18 ± 3.07	10.57 ± 3.79	–1.113	0.268
Age of onset (years)	66.18 ± 5.20	65.48 ± 4.43	–0.525	0.601
Disease duration (months)	5.33 ± 5.27	8.05 ± 7.61	1.622	0.11
With or without etiology (*n*, %)	24 (61.5%)	15 (71.4%)	0.587	0.444
**Types of antidepressant drugs**				
Escitalopram (*n*, %)	21 (53.8%)	15 (71.4%)	1.758	0.185
Sertraline (*n*, %)	18 (46.2%)	6 (28.6%)		
Antidepressant dosage (mg/day)				
Escitalopram	13.81 ± 4.445	16.33 ± 4.419	–1.684	0.101
Sertraline	102.78 ± 3.907	116.67 ± 18.960	1.422	0.169
HAMD-17	21.52 ± 3.53	21.79 ± 3.53	–0.284	0.778

*TE, treatment effective group; TI, treatment ineffective group; HAMD, Hamilton Depression Rating Scale.*

There was no significant difference of baseline TMT and RBANS (all *p* > 0.05) between TE and TI patients. All differences in the change of TMT and RBANS after treatment were not statistically significant between TE and TI patients (all *p* > 0.05). Repeated-measure ANCOVA analysis showed that there was no significant time and group interaction for the TMT and RBANS score between TE and TI patients (all *p* > 0.05).

### Predictors of Baseline Cognitive Performance on Efficacy in Late-Life Depression Group

**FIGURE 1:**
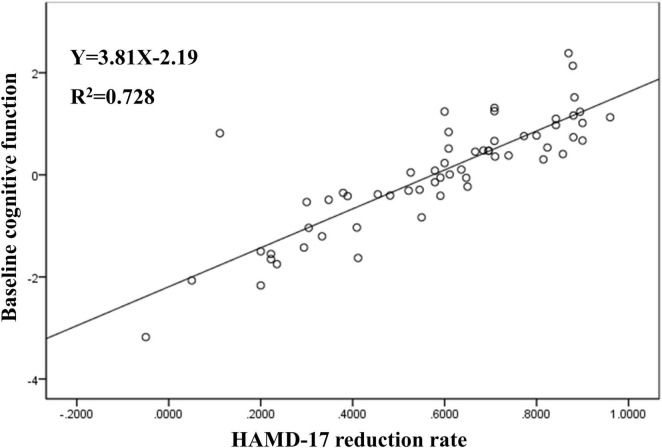
Predictors of the cognitive function on HAMD-17 reduction rate in LLD.

Multiple linear regression analysis showed that the change in HAMD-17 score after treatment was significantly correlated with baseline score of picture naming (B = 0.043; *p* = 0.04), figure copy (B = −0.025; *p* = 0.004), and digit span (B = 0.028; *p* = 0.027) in the LLD group (see Table 4 and Figure 1).

**TABLE 4:** Multiple linear regression analysis of predictors of antidepressant efficacy in LLD.

Variable	B	Std. error	Beta	*t*-value	*p*-value
(Constant)	0.196	0.202		0.973	0.335
Picture naming	0.043	0.02	0.269	2.101	0.04
Figure copy	–0.025	0.008	–0.397	–2.98	0.004
Digit span	0.028	0.012	0.325	2.282	0.027

*LLD, late life depression.*

**FIGURE 2:**
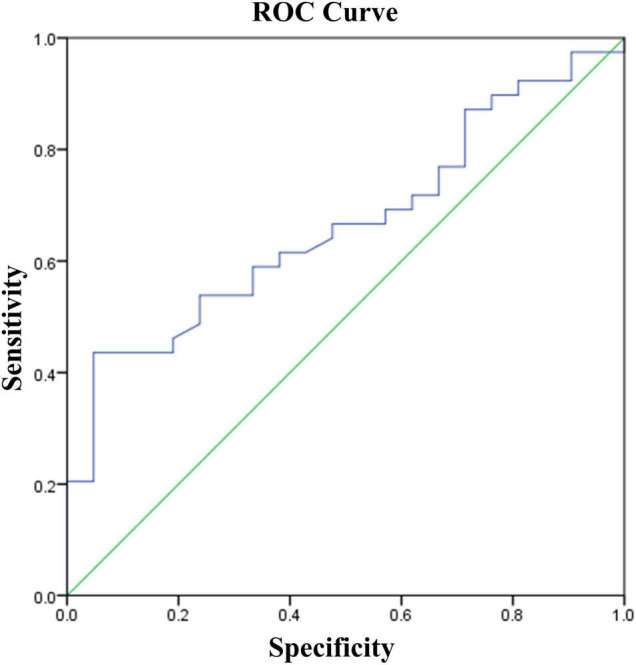
Prediction model of treatment effectiveness of LLD.

Binary logistic regression analysis was performed with response or non-response (TE = 1, TI = 0) as the dependent variable and the baseline cognitive function including TMT and RBANS scores as independent variables. The results showed that the higher of the delayed memory score, the better of the efficacy (OR = 1.107, 95% confidence interval: 1.026–1.199, *p* = 0.009). The area under the ROC curve suggested that the predicting efficacy of delayed memory was 0.665 (95% confidence interval: 0.529–0.802, *p* = 0.036), and the sensitivity was 0.436, specificity was 0.952, and Youden index was 0.388 when the optimal cut-off value was taken (see Figure 2).

## Discussion

In this study, first-episode LLD patients treated with 8-week of escitalopram or sertraline demonstrated improvement of depression and partial cognitive function including immediate memory, language, and delayed memory. Patients with lower level of baseline cognitive function, including figure copy, picture naming, digital span, delayed memory had poorer response after 8-week treatment. This finding suggested that cognitive function may be a predictor of 8-week antidepressant treatment outcome.

The results of this study showed that there was broad cognitive function impairment in first-episode, drug naïve LLD patients and supports evidence from previous observations. Recent studies have found that cognitive deficits are a core feature of depression in elderly, and cognitive complaints in older adults with depression include learning difficulties, slow processing, and executive dysfunction (13, 27).

Digital span consisted of digit span forward and digit span backward. Digit span forward was used to assess working memory (28). Working memory involves holding information in mind and mentally working with it (29). A deficit in working memory may correlate with functional difficulties in maintaining mental set in the face of distracting affective input during depressive episode. A positive relationship between digit span forward and treatment remission was reported that LLD patients with poorer working memory performance was slower to remit at the end of treatment (15). Another study had demonstrated that poorer working memory was associated with poorer response at 4 weeks fluoxetine treatment in 72 youth depressed patients (30). Furthermore, this study demonstrated that poor working memory was related with worse efficacy in LLD patients.

Digit span backward was used to assess sustained attention. Shiroma et al. (31) found that people with good concentration at baseline were more likely to better treatment respond. Etkin et al. (20) analyzed predictors of efficacy in patients with youth depression (*n* = 1,008; 665 completers) and found that impaired attention was associated with antidepressant efficacy. This study also supported that attention was a predictor of response to antidepressant treatment.

This study confirms that figure copy was also correlated with treatment response. Figure copy was used to assess visuospatial function including stereopsis vision. Stereopsis vision with binocular disparity mainly through the three-dimensional reconstruction of depth-related information in the visual cortex, and stereopsis was considered a visual perception that may affect cognitive-related tasks, including visual memory, visual attention (32). A previous large scale retrospective study reported that visuospatial was significantly related to eventual reduction of depression severity in youth depression patients (33). Another finding was that youth depression patients obtained rehabilitation of psychosocial function by improving cognitive ability in spatial structure (34). This study suggested that visuospatial also played an important role in predicting the efficacy of LLD patients.

We also observed that picture naming and delayed memory could be serve as a significant predictor of treatment response. Picture naming was used to reflect language function. Several studies suggested that language function has the potential to predict antidepressant efficacy in geriatric depression (18, 35). A study found that 25 patients with depression performed significantly worse delayed memory than non-responders after a single injection of ketamine (36). Decreased verbal function may have a “top-down” negative impact on verbal episodic memory performance and may predict remission rates in geriatric depression (35, 37).

Cognitive impairments in LLD maybe related with the alteration in neuroimaging. Cognitive impairment in LLD may be associated with cerebral abnormalities in the prefrontal, medial prefrontal, and parietal cortex (38). Imaging studies of LLD identified microstructural abnormalities in white matter tracts that connect the prefrontal cortex with subcortical and posterior cortical regions, which have been linked to cognitive dysfunction (10, 39). Low white matter integrity in distributed networks tracts supporting executive function was associated with poor response in LLD patients (10). Functional neuroimaging study has shown that the increased functional connectivity of the left dorsolateral prefrontal cortex and bilateral prefrontal regions was associated with the severity of depression and executive function and working memory in LLD (40). Memory impairment is typical of medial temporal region involvement, especially where hippocampal atrophy has been found in LLD (41). Several reports have shown that decreased cognitive task-related activity in the prefrontal cortex in LLD prior to treatment, which is normalized following treatment (42). Increased cognitive control network functional connectivity and decreased default mode network functional connectivity were observed in LLD with remission, but not in patients with ineffective treatment (43). The functional connectivity between cingulate cortex and ventromedial prefrontal lobe in default mode network, and between dorsal anterior cingulate gyrus and insular lobe in Salience Network was enhanced in LLD with treatment response (44). It could be deduced that the mechanism of cognitive impairment maybe overlapped with the pathophysiology of efficacy. Cognitive function would be used as a potential biomarker for efficacy prediction in LLD patients.

There were several limitations in this study. First, the sample size was relatively small. Second, the drop-out rate (25%) was relatively high which limited the exploration of the trajectory of cognitive ability changes between TE and TI group. Third, participants visited only twice (baseline and after 8-week treatment) which limited the investigation of group difference in cognitive function at early phase of treatment. The results of this pilot study should be interpreted with caution. Future study may need to recruit larger sample size of participants, combine more cognitive indicators and frequent follow-up visits to find a prediction model with high sensitivity and specificity.

## Conclusion

This study found that lower level of baseline cognitive function had poorer antidepressant response after 8-week treatment in LLD patients. Cognitive function may be used as a predictor of antidepressant treatment outcome, especially working memory, attention, visuospatial, and language function. The results of this pilot study should be interpreted with caution because of the small sample size. Further studies using larger sample sizes are needed to assess these preliminary results.

## Data Availability Statement

The raw data supporting the conclusions of this article will be made available by the authors, without undue reservation.

## Ethics Statement

The studies involving human participants were reviewed and approved by the Ethics Committee of Beijing Anding Hospital, Capital Medical University. The patients/participants provided their written informed consent to participate in this study.

## Author Contributions

WP contributed to study design, data analysis, investigation, and writing—original draft. WP and DZ contributed to prescribing and administering antidepressant drugs, depression rating, and cognitive text. CL and YL contributed to patients recruitment and consenting, analyzed and interpreted the data, and revised the manuscript. PM contributed to funding acquisition, project administration, writing—review, and editing. YR and XM contributed to conceptualization, methodology, supervision, and writing—review and editing. All authors contributed to the article and approved the submitted version.

## Conflict of Interest

The authors declare that the research was conducted in the absence of any commercial or financial relationships that could be construed as a potential conflict of interest. The handling editor LL declared a shared parent affiliation with the authors at the time of review.

## Publisher’s Note

All claims expressed in this article are solely those of the authors and do not necessarily represent those of their affiliated organizations, or those of the publisher, the editors and the reviewers. Any product that may be evaluated in this article, or claim that may be made by its manufacturer, is not guaranteed or endorsed by the publisher.
